# Extracellular Vesicles in Fungi: Past, Present, and Future Perspectives

**DOI:** 10.3389/fcimb.2020.00346

**Published:** 2020-07-15

**Authors:** Juliana Rizzo, Marcio L. Rodrigues, Guilhem Janbon

**Affiliations:** ^1^Unité Biologie des ARN des Pathogènes Fongiques, Département de Mycologie, Institut Pasteur, Paris, France; ^2^Instituto Carlos Chagas, Fundação Oswaldo Cruz, Curitiba, Brazil; ^3^Instituto de Microbiologia Paulo de Góes, Universidade Federal Do Rio de Janeiro (UFRJ), Rio de Janeiro, Brazil

**Keywords:** extracellular vesicles, fungal infections, fungal physiology, intercellular communication, pathogenesis

## Abstract

Extracellular vesicles (EVs) have garnered much interest in the cell biology and biomedical research fields. Many studies have reported the existence of EVs in all types of living cells, including in fifteen different fungal genera. EVs play diverse biological roles, from the regulation of physiological events and response to specific environmental conditions to the mediation of highly complex interkingdom communications. This review will provide a historical perspective on EVs produced by fungi and an overview of the recent discoveries in the field. We will also review the current knowledge about EV biogenesis and cargo, their role in cell-to-cell interactions, and methods of EV analysis. Finally, we will discuss the perspectives of EVs as vehicles for the delivery of biologically active molecules.

## Introduction

Extracellular vesicles (EVs) is a general term used to describe cell-derived double-layer phospholipid membrane particles that reach the extracellular environment (van Niel et al., 2018). These membranous particles are released by cells in all the three domains of life (Gill et al., 2019), and many studies have highlighted their relevance in diverse biological contexts (Maas et al., 2017; van Niel et al., 2018; Bielska and May, 2019; Rybak and Robatzek, 2019; Palacios et al., 2020). EVs are heterogeneous in biogenesis pathways, size, cargo, membrane composition, and biological functions. They participate in many cellular physiological events, including disease development in humans and animals (Shah et al., 2018; Xu et al., 2018). The recent advances in this still emerging field have contributed to the construction of solid knowledge that is rapidly evolving to the formulation of applied tools, including drug delivery systems and vaccine prototypes.

Based on their biosynthetic pathways, mammalian EVs are usually categorized in two broad classes: microvesicles (also called ectosomes or microparticles) and exosomes (van Niel et al., 2018; Latifkar et al., 2019; Mathieu et al., 2019). Microvesicles are generally larger vesicles, from 50 nm to 2,000 nm in diameter, that are formed by the direct outward budding of the plasma membrane. Exosomes are smaller EVs ranging from 30 to 150 nm in diameter that are produced by the endosomal pathway. The biogenesis of exosomes starts when endosomes maturate to form multivesicular bodies (MVBs). These structures fuse with the plasma membrane and release their luminal vesicles to the outer space (van Niel et al., 2018; Latifkar et al., 2019). The exosome population is apparently more complex than initially thought. Recently, by employing an asymmetric-flow field-flow fractionation analysis of melanoma-derived exosomes, two exosome subpopulations were identified, including a large subset ranging from 90 to 120 nm, and a small subset from 60 to 80 nm (Zhang et al., 2018). These subsets of nanoparticles were shown to be packed with different cargo, highlighting the diversity of particles secreted by living cells and opening new questions on their biogenesis and functions (Zhang et al., 2018; Mathieu et al., 2019).

In fungi, since their first description in 2007 (Rodrigues et al., 2007), EVs have been identified in twenty different species, comprising yeast and filamentous fungi. Despite the increasing number of studies on EVs, we still have limited information on their structural properties, biogenesis, and functional outcomes. In this review, we will discuss early reports suggesting the existence of fungal EVs. We will then move our discussion to recent insights, technical hurdles, and the relevance of EVs for fungal biology and intercellular communication during interaction with different host cells.

## Extracellular Vesicles in Fungi: Historical Aspects and Overview of Recent Discoveries

Studies in the early 1970s suggested the existence of fungal EVs in different models. In 1972, Gibson & Peberdy analyzed the ultrastructure of *Aspergillus nidulans* protoplasts and described a “region of protoplast plasmalemma exhibiting outpushing,” which lead to the production of outer membranous particles, once called “subprotoplasts” (Gibson and Peberdy, 1972). Another example of microscopical evidence of the existence of fungal EVs was provided in 1973 by Takeo and collaborators (Takeo et al., 1973). They reported the presence of “spherical invaginations which secrete the vesicles outside the cell membrane” in *Cryptococcus neoformans* (Takeo et al., 1973). In 1977, ”extracellular vesicles” was used for the first time in the fungal literature by Chigaleichik and colleagues during the analysis of extracellular lipid structures of *Candida tropicalis* cultivated in the presence of n-alkanes (Chigaleichik et al., 1977).

In 1990, “membrane-bound vesicles which traverse the wall through specialized pimple structures” were reported in *Candida albicans* (Anderson et al., 1990). Eight years later, studies on the cell wall dynamics of *Schizosaccharomyces pombe* demonstrated that protoplasts under cell wall regeneration manifested an increased number of secretory vesicles, including vesicle-like particles in the outer space (Osumi, 1998). In the same study, particles at the *C. albicans* cell surface, at that time called “warty projections,” were also reported (Osumi, 1998). In 2000, membrane formations across the periplasmic space, linking the plasma membrane to the inner face of the cell wall, were reported in *C. neoformans*, suggesting the occurrence of vesicular traffic across the fungal cell wall (Rodrigues et al., 2000). Noteworthy, it is very likely that other reports similarly suggested vesicle-like particles in the outer space of fungi, but to our knowledge, these studies compose the first set of experimental evidence suggesting the existence of fungal EVs.

The first study directly focusing on fungal EVs was published in 2007 in *C. neoformans*. Fungal EVs were proposed to be the vehicles for polysaccharide export across the fungal cell wall (Rodrigues et al., 2007). Many subsequent studies demonstrated EV production in yeast forms of *C. gattii, Histoplasma capsulatum, C. albicans, C. parapsilosis, Sporothrix schenckii, S. brasiliensis, Paracoccidioides brasiliensis, P. lutzii, Malassezia sympodialis, Saccharomyces cerevisiae, Pichia fermentans*, and *Exophiala dermatitidis* (Albuquerque et al., 2008; Gehrmann et al., 2011; Vallejo et al., 2011; Vargas et al., 2015; Leone et al., 2017; Bielska et al., 2018; Ikeda et al., 2018; Peres Da Silva et al., 2019; Lavrin et al., 2020).

**Figure 1:**
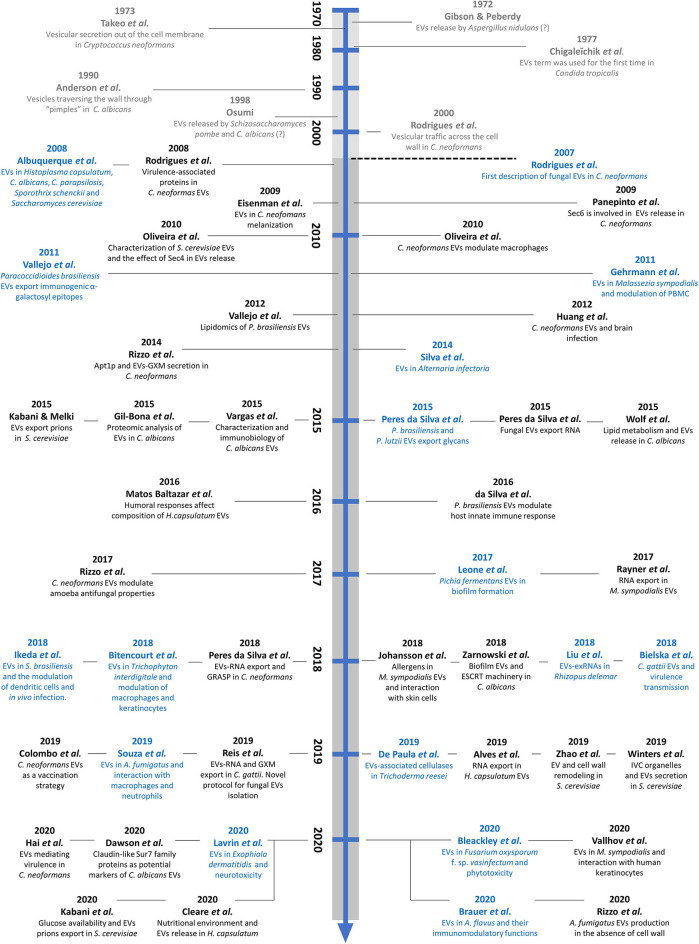
Timeline showing early evidence and recent discoveries in the field of fungal EVs. The studies in gray represent early suggestions of fungal EVs. The dashed line represents the first direct description of fungal EVs, in the *C. neoformans* model. The studies in blue illustrate the initial characterization of EVs in the twenty different fungal species, and those in black represent compositional, methodological, or functional discoveries regarding fungal EVs.

Compared to yeasts, little is known about EVs in filamentous fungi. However, their presence has been described in different species, including *Trichoderma reesei*, a fungus involved in lignocellulosic degradation (de Paula et al., 2019), in the phytopathogens *Alternaria infectoria* (Silva et al., 2014) and *Fusarium oxysporum* f. sp. *vasinfectum* (Bleackley et al., 2019b), and in the dermatophyte *Trichophyton interdigitale* (Bitencourt et al., 2018). In human filamentous pathogens, EVs have been described in the emerging pathogen *Rhizopus delemar* (Liu et al., 2018) and in the major common causative agents of invasive aspergillosis, *A. fumigatus and A. flavus* (Souza et al., 2019; Brauer et al., 2020; Rizzo et al., 2020). A timeline pointing point out the historical aspects and the recent discoveries of fungal EVs is presented in Figure 1.

## Biogenesis, Selection of Cargo and Release of EVs in Fungi

The processes regulating fungal EV biogenesis and the specificity of cargo remain unresolved, and most of our hypotheses come from mammalian studies. Fungal EVs are carriers of proteins, lipids, nucleic acids, polysaccharides, toxins, allergens, pigments, and even prions, as recently reviewed (Bleackley et al., 2019a; De Toledo Martins et al., 2019). Many of these molecules are associated with fungal physiological aspects, such as metabolism and cell wall biogenesis, but also with stress responses, antifungal resistance and pathogenesis (Rodrigues et al., 2007, 2008; Albuquerque et al., 2008; Eisenman et al., 2009; Vallejo et al., 2012a,b; Gil-Bona et al., 2015; Kabani and Melki, 2015; Peres Da Silva et al., 2015b; Vargas et al., 2015; Zarnowski et al., 2018; Alves et al., 2019; Zhao et al., 2019).

By analogy with metazoan counterparts, it has been suggested that the release of fungal EVs and selection of cargo can require diverse secretory routes, including regulators of conventional and unconventional secretory pathways (Oliveira et al., 2013; Bielska and May, 2019; Silva et al., 2019). Among the conventional secretory regulators, the Sec6 protein, involved in the exocytosis of post-Golgi secretory vesicles to the plasma membrane, was reported to be associated with EV release in *C. neoformans*. A reduction of EV release level in a sec6 mutant strain was associated with the impaired secretion of virulence-associated molecules (Panepinto et al., 2009). Additionally, mutation of *SEC4*, which encodes a Rab family GTPase; essential for vesicle-mediated exocytic secretion and autophagy, altered EV composition and the kinetics of extracellular release in *S. cerevisiae* (Oliveira et al., 2010b). The Sec1 protein, which is involved in the fusion of Golgi-derived exocytic vesicles with the plasma membrane, also participated in EV composition, but *SEC1* deletion did not affect EV release (Oliveira et al., 2010b). These studies suggest a key role for the Golgi-derived secretory pathway in the vesicular trans-cell wall traffic.

Among the regulators of unconventional secretion, Oliveira and collaborators suggested that the Golgi reassembly stacking protein (GRASP) was involved in EV release in *S. cerevisiae* (Oliveira et al., 2010b). GRASP was recently shown to participate in EV-mediated export of mRNA in *C. neoformans* (Peres Da Silva et al., 2018) and is an important regulator of *C. neoformans* virulence (Kmetzsch et al., 2011). In addition to GRASP, members of the Endosomal Sorting Complex Required for Transport (ESCRT) machinery involved in the formation and functionality of MVBs, such as the Snf7 and Vps23 proteins, affected EV protein profile in *S. cerevisiae* (Oliveira et al., 2010b).

It was also demonstrated by Zhao and colleagues in *S. cerevisiae*, that mutation of the *VPS2, VPS23*, and *VPS36* genes, which encode components of the ESCRT machinery, affected EV proteomic profile and EV abundance (Zhao et al., 2019). EVs produced by ESCRT mutants showed enrichment in cell wall remodeling enzymes, including the glucan synthase subunit Fks1 and the chitin synthase Chs1 (Zhao et al., 2019). Similar results were obtained in *C. albicans*, in which mutations in the subunits of the ESCRT machinery resulted in decreased EV production, in comparison with wild-type strains (Zarnowski et al., 2018). It was recently shown that the lack of another protein of ESCRT complex, Vps27, resulted in the accumulation of MVB structures and release of enlarged EVs in *C. neoformans*. This phenotype was associated with an impaired laccase trafficking to the cell wall (Park et al., 2020). Since EVs were already reported to contain laccase (Rodrigues et al., 2008), this result provides further insights into laccase-associated EV transport and virulence in *C. neoformans*.

Additional mutations have been shown to affect EVs production or cargo composition, although it remains to be known whether these phenotypes are their direct or indirect consequences of these mutations. For instance, the deletion of the lipid flippase encoding gene *APT1* has been reported to alter EV size, EV-mediated secretion of the major capsular polysaccharide, glucuronoxylomannan (GXM), as well as virulence in *C. neoformans* (Rizzo et al., 2014). In *C. gattii*, the deletion of *AIM25* encoding a putative lipid scramblase was reported to result in the production of larger EVs and with altered RNA content, despite the normal EV-associated GXM content (Reis et al., 2019). These data suggest that lipid transporters, and perhaps other regulators of lipid metabolism, could play an important role in biogenesis and cargo selection of fungal EVs. Indeed, lipid biosynthetic genes such as the phosphatidylserine decarboxylase encoding genes (*PSD1* and *PSD2*) in *C. albicans* were reported to influence EV size and protein composition, suggesting an association between lipid metabolism and composition of EVs (Wolf et al., 2015).

It has also been suggested that other regulators of fungal cell physiology could play a role in the release of EVs. For instance, a *C. neoformans* mutant lacking a putative G_1_/S cyclin gene had increased production of EVs (Garcia-Rodas et al., 2014). Chitin synthase genes (CHS) were also suggested to play a role in vesicular release, as inferred from the observation that the deletion of chitin synthases genes in *C. neoformans* resulted in a significantly reduced release of EVs (Rodrigues et al., 2018). Recently, it was also shown that *S. cerevisiae* cytoplasmic organelles, called intracellular vesicle clusters (IVCs), serve as sites for the synthesis and selection of EV-associated proteins tagged for secretion (Winters et al., 2020).

Although the studies above provided relevant information on potential mechanisms of EV production in fungi, the key genetics and transcriptional networks, and eventually the post-translational processes underlying fungal EV production and selection of cargo, are unknown. EV release is influenced by fungal lifestyle and modulated by environmental or host-posed conditions (Eisenman et al., 2009; Matos Baltazar et al., 2016; Baltazar et al., 2018; Zarnowski et al., 2018). Additionally, externally added compounds such as EDTA, molecules produced by mammalian cells, such as Galectin-3 (Gal-3) or serum albumin, were reported to reduce vesicular release, or lead to EV disruption (Robertson et al., 2012; Wolf et al., 2012; Almeida et al., 2017). These studies highlight the complex mechanisms associated with EV formation and release, including their extracellular stability.

## EVs and Cell Wall Crossing

The presence of a thick cell wall was historically considered as a barrier for the outward transition and release of membrane-derived vesicles, as recently discussed (Coelho and Casadevall, 2019). This view contrasts with early studies demonstrating that the fungal cell wall contains several major lipids, which could be components of transitory membrane structures associated with trans-cell traffic (Kanetsuna et al., 1969; Domer, 1971; Cox and Best, 1972). The idea of the cell wall as a friendly environment for lipids was consolidated by numerous reports on membranous structures in association with the cell wall and in culture supernatants. Consequently, several hypotheses have been proposed to understand how the cell wall structure is compatible with the transit of lipid membranes. There are three non-mutually exclusive hypotheses that related to this point. First, vesicles could move across the cell wall through a guide channel. Second, cell wall remodeling enzymes could generate areas facilitating EV transit. Finally, turgor pressure could force vesicles to pass through cell wall pores (Wolf and Casadevall, 2014; Brown et al., 2015).

In *C. neoformans*, Wolf and collaborators used electron microscopy techniques to identify single and multiple vesicle-like particles directly in the cell wall, without any apparent trans-cell wall channel or changes in vesicle surroundings, which argues against the presence of channels for vesicle release in fungi (Wolf et al., 2014). It was also demonstrated that liposomes containing amphotericin B (AmBisome) ranging from 60 to 80 nm in diameter crossed the fungal cell wall from outside to the intracellular space and reached the plasma membrane in their intact form, even though the predicted porosity of the cell wall was too small (pore size around 5.8 nm) to allow their transit (Walker et al., 2018). After these observations, Walker and colleagues described yeast cell walls as viscoelastic structures, permeable to membranous particles.

Recently, it was also shown that EVs from *S. cerevisiae* contain cell wall-related proteins, including enzymes that participate in the degradation and reorganization of polysaccharides, suggesting a role in cell wall remodeling (Zhao et al., 2019). Additionally, it was recently shown that wall-less *A. fumigatus* cells export plasma membrane-derived EVs containing a complex combination of proteins and glycans. EVs produced by germinating conidial protoplasts increased in number and differed in cargo when the cells were incubated under cell wall regeneration conditions (Rizzo et al., 2020). Finally, another recent study demonstrated that polymorphonuclear granulocytes produce EVs that associate with the cell wall of *A. fumigatus* and even enter fungal hyphae, resulting in alterations in the morphology of the fungal cell wall (Shopova et al., 2020). These studies highlight the cell wall as a dynamic structure with flexible viscoelastic properties and provide new insights into how vesicles can cross the cell wall to reach the extracellular space. Clearly, these studies also open questions on how EVs can play a role in cell wall biosynthetic processes and inter-kingdom communication as signaling entities.

## Cell-to-Cell Communication Mediated by EVs

The process of cell wall crossing by EVs has functional consequences in recipient cells (Regente et al., 2017; Bielska et al., 2018; Cai et al., 2018; Rodrigues and Casadevall, 2018). Therefore, here we will explore the literature on EV-mediated communication between fungal cells and in the bidirectional cross-talk between fungi and other organisms.

The relevance of fungal EVs during cellular interaction at the community level, in different stages of fungal lifecycle or even transferring virulence-associated molecules from one strain to another, has been suggested. For instance, it was recently described that EVs produced in *C. albicans* biofilms are different from those produced by free-living planktonic cells, and the release of EVs is an important feature for the proper biofilm formation and drug resistance (Zarnowski et al., 2018). Previously, in the dimorphic yeast *P. fermentans* it was also suggested that EVs could play an active role during the dimorphic transition in response to the growth conditions, including biofilm formation (Leone et al., 2017). Both studies suggest that EVs could participate in intercellular communication during biofilm formation, stimulating studies on the relationship of other microbial biofilms with fungal EVs.

Other examples of cell-to-cell communication mediated by fungal EVs came from studies on *Cryptococcus*. It was previously described that the Vancouver Island outbreak lineage of *C. deuterogattii* display an increased ability to proliferate inside host macrophages through a mechanism called “division of labour” (Voelz et al., 2014). During this process, cells coordinate their behavior to increase the intracellular proliferation of the population as a whole (Voelz et al., 2014). This process has been recently shown to be regulated by EVs (Bielska et al., 2018). EVs obtained from this outbreak lineage were internalized by macrophages pre-infected with cells from a non-outbreak lineage and trafficked to the phagosome, inducing a rapid intracellular proliferation of the non-outbreak fungal cells. This process seems to be restricted to intra-species communication since EVs purified from a virulent strain of *C. neoformans* did not result in the same outcome, even at the highest concentrations of EVs (Bielska et al., 2018).

Very recently, Hai et al. reported that sterile culture filtrates from highly virulent VNIa-5 strains of *C. neoformans* isolated from immunocompetent patients, but not from HIV patients, promoted an increase in the pathogenic potential of less virulent VNIa-5 isolates. This process probably required EV-associated proteins (Hai et al., 2020). These results open new avenues on how EVs can act in virulence transfer in many different contexts, such as in fungal co-infections.

The complexity of the relationship of EV cargo and their functions in intercellular communication was reinforced by the discovery that fungal EVs contain prions and cell wall remodeling enzymes (Kabani and Melki, 2015; Zhao et al., 2019). In *S. cerevisiae*, it was demonstrated that the fungal prion Sup35p was exported via EVs both in its soluble and aggregated infectious states (Kabani and Melki, 2015). Considering that prions are transmitted vertically to the progeny or horizontally during mating, it is reasonable to suggest that EVs could mediate vertical and horizontal transfer of prions-like protein in fungi (Kabani and Melki, 2016; Zhao et al., 2019). Still in *S. cerevisiae*, it was recently shown that EVs can be taken up by fungal cells and play a critical role in cell wall remodeling. Fungal EVs containing cell wall associated enzymes, such as glucan and chitin synthases, were able to enhance yeast cell viability upon cell wall stress, induced by the presence of the 1,3-β-glucan synthase inhibitor antifungal drug, caspofungin (Zhao et al., 2019). This latter data raises the question if the drug resistance could also happen in a community level basis and bolster fungal infection processes, as previously discussed for drug resistance in *C. albicans* biofilms (Zarnowski et al., 2018).

In addition to what is known about communication between fungal cells, several studies on the relevance of fungal EVs in cellular communication with mammalian cells are available in the literature, as previously reviewed (Zamith-Miranda et al., 2018; Bielska and May, 2019; Freitas et al., 2019; Silva et al., 2019). In all morphological stages of many pathogens, fungal EVs were shown to be internalized by mammalian cells in processes that culminated with the modulation of antimicrobial activities and diverse immunogenic responses, including the activation of pro-inflammatory and anti-inflammatory cytokines. In these experiments, host cells interacting with EVs comprise murine macrophages (including bone marrow-derived macrophages), dendritic cells, and neutrophils, in addition to human peripheral blood mononuclear cells, keratinocytes, monocytes, macrophages, and brain microvascular endothelial cells (Oliveira et al., 2010a; Gehrmann et al., 2011; Vallejo et al., 2011; Huang et al., 2012; Peres Da Silva et al., 2015a; Vargas et al., 2015; Da Silva et al., 2016; Bielska et al., 2018; Bitencourt et al., 2018; Ikeda et al., 2018; Johansson et al., 2018; Souza et al., 2019; Vallhov et al., 2020).

Recently, it was shown that melanized-EVs obtained from the extremophilic fungus *Exophiala dermatitidis* were able to strongly affect the viability of human neuroblastoma cells, while non-melanized EVs were considerably less neurotoxic (Lavrin et al., 2020). These data demonstrated that EV cargo is relevant for their biological effect on host cells, and strengthen the notion that fungal EVs enclose diverse virulence-associated molecules, such as melanin, as previously reported for *C. neoformans* (Rodrigues et al., 2008; Eisenman et al., 2009).

Several elements can interfere in EV-mediated interactions between fungi and mammalian cells. Baltazar and collaborators showed that the binding of monoclonal antibodies to *H. capsulatum* modulated vesicle composition at both quantitative and qualitative levels, leading to diverse immune effector mechanisms (Matos Baltazar et al., 2016; Baltazar et al., 2018). Reales-Calderon and colleagues also showed that macrophage-derived EVs change their size and protein composition in response to *C. albicans* infection, suggesting a role of host cells EVs in fungi-macrophage communication (Reales-Calderon et al., 2017). Recently, it was also shown *A. fumigatus* cells triggered EV release by human neutrophils. EVs released by neutrophils exposed to conidia had antifungal properties and inhibited the growth of *A. fumigatus* hyphae. The same outcome was not observed for EVs released by uninfected neutrophils (Shopova et al., 2020). These data suggest that EV release by mammalian cells represent a still unexplored mechanism of antifungal defense during host-pathogen interactions.

Besides regulating the responses of mammalian cells, fungal EVs were also described to modulate the physiology of environmental predators, including *Acanthamoeba castellanii* (Rizzo et al., 2017). During the fungi-amoebae interaction, EVs released by *C. neoformans* were internalized by *A. castellanii* with no impact to the predator's viability. EVs modulated amoebal antifungal properties by inducing enhanced yeast intracellular survival. The same effect did not occur when amoebae were treated with the capsular polysaccharide GXM (Rizzo et al., 2017). Although *A. castellannii* was also shown to release EVs (Goncalves et al., 2018), the impact of amoeba EVs on fungal biology remains to be elucidated.

EVs also participate in plant-fungi communication (Cai et al., 2019). In 2011, based on the ultrastructural characterization of the haustorium-forming phytopathogen *Golovinomyces orontii*, it was observed MVB-like structures fusing with (or budding off from) the fungal plasma membrane and also membrane-bound vesicles in the extra-haustorial matrix, suggesting the occurrence of vesicular release outside of the haustorial cell wall during plant-fungi interactions (Micali et al., 2011). Later on, EVs from sunflowers were shown to be internalized by the fungus *Sclerotinia sclerotiorum*, a phytopathogen able to infect numerous host plants and cause severe rot (Regente et al., 2017). Plant-derived EVs were enriched in cell wall remodeling enzymes and defense proteins. Once in contact with fungal cells, plant EVs caused morphological changes, impairment of growth and cell death, which led to the hypothesis that EVs could function as vehicles for the delivery of components involved in plant defense mechanisms against fungal infections (Regente et al., 2017).

Cai and collaborators demonstrated that *Arabidopsis* cells release EVs containing siRNAs that were efficiently taken-up by the fungus *Botrytis cinerea*, which resulted in the silencing of virulence-associated fungal genes (Cai et al., 2018). The mechanism behind this observation involved the silencing of fungal virulence-associated genes through mRNA cleavage (Cai et al., 2018). *Arabidopsis* EVs were also proposed to deliver siRNAs into the plant pathogen *Phytophthora capsici*, possibly contributing to host-induced gene silencing during natural infection (Hou et al., 2019).

EVs were also involved in arbuscular mycorrhizal symbioses (Roth et al., 2019), as concluded from the accumulation of EVs in the contact area of fungi with plant cells. It is not known if these EVs are originated from plant and/or fungal cells, but their detection in the interaction interface suggests that these membranous particles could be mediating the cell-to-cell communication between the two symbionts. Although these studies highlight the relevance of EVs in mediating molecule exchange from plant to fungi, an unanswered question is whether fungi use EVs to deliver effector molecules to plants during mutualistic or parasitic relationships. Accordingly, in EVs from the cotton pathogen *Fusarium oxysporum* f. sp. *vasinfectum* were described to induce a phytotoxic response in plants (Bleackley et al., 2019b), which reinforce this hypothesis. This subject has been discussed in detail in recent reviews (Huang et al., 2019; Kwon et al., 2020).

## Methods of Purification and Technical Hurdles of EVs Characterization

The difficulties posed by EV isolation methods, quantification, imaging, and functional analysis are technical hurdles that challenge the field of EV research (Margolis and Sadovsky, 2019). The debate concerning the principles for EV isolation, characterization of cargo and biological functions has motivated numerous consortiums in the field of mammalian EVs to establish collaborative projects directed to the development of robust methods for isolation, analysis, interpretation and reproducibility of experiments (Consortium et al., 2017; Thery et al., 2018; Das et al., 2019).

Despite substantial progress, research on fungal EVs is still in its infancy (Bielska and May, 2019). In this context, it is essential to point out the limitations and advances of the investigation of fungal EVs, including the experimental models of EV analysis. The regular protocols that have been used in the past decade to isolate fungal EVs are time-consuming and rely on handling liters of culture supernatant using low-speed centrifugation, followed by filtration, volume concentration, and collection of EV-rich fractions by ultracentrifugation (Rodrigues et al., 2016). This general protocol has been used since the first description of EVs in *C. neoformans (Rodrigues et al.*, *2007**)*, but there are serious limitations, including low yield, possible co-isolation of non-vesicular extracellular molecules, and the isolation of mixed EV populations.

The recent use of an asymmetric flow field-flow fractionation analysis revealed a previously unknown population of particles smaller than 50 nm (around 35 nm), named exomeres. These structures lack the external membrane and, therefore, are considered as non-vesicular nanoparticles that can be co-isolated with exosomes (Zhang et al., 2018; Mathieu et al., 2019). Exomeres have not been documented in fungi so far. Therefore, one can speculate that their presence in fungal secretomes could also represent a contamination of EV fractions. The hurdles of isolation and characterization of homogeneous subgroups of EVs have been a matter of debate in the EV field as a whole, and recent calls for protocol improvements and accurate analysis have been published (Thery et al., 2018; Raposo and Stahl, 2019).

Different approaches have been suggested to increase the purity, yield, and functional potential of mammalian cells EV purification protocols (Mateescu et al., 2017; Thery et al., 2018; Takov et al., 2019). In fungi, density gradient ultracentrifugation with sucrose or iodixanol was successfully used for EV fractionation (Rodrigues et al., 2007; Oliveira et al., 2009; Kabani and Melki, 2015; Rayner et al., 2017; Bleackley et al., 2019b). Also, size-exclusion chromatography after clarification and ultracentrifugation of culture supernatants was used to isolate EVs from *C. albicans* biofilms (Zarnowski et al., 2018). However, the main disadvantage of applying additional purification steps during EV isolation is the decrease in the purification yield (Chutkan et al., 2013).

An alternative approach for the isolation and analysis of fungal EVs has been recently described. Reis and collaborators have demonstrated that isolating EVs from cells growing on solid media had many advantages in comparison to the previously used protocol in liquid medium (Reis et al., 2019). The optimized protocol was shown to be faster, with higher yields, and applicable to the biological evaluation of EVs (Reis et al., 2019). Nonetheless, all the protocols used so far end up in the generation of EV-rich centrifugation pellets, which consistently comprise heterogeneous populations, with varying sizes and physical-chemical properties. These latter aspects make EVs subpopulations indistinguishable, thus lessening the accuracy of their characterization, which can culminate in misleading interpretations of functional roles (Raposo and Stahl, 2019).

The analysis of the dimension of fungal EVs has been predominantly based on three different technical approaches: dynamic light scattering (DLS), nanoparticle tracking analysis (NTA), and electron microscopy (EM) (Bielska and May, 2019; Palacios et al., 2020). A combination of these methods and others certainly improves accuracy. In this sense, NTA proved to be a useful method to evaluate the distribution of EV subtypes on different fungal species, in association with particle quantification (Reis et al., 2019). DLS provides similar results, with the limitation of not being quantitative. However, considering that DLS and NTA techniques are based on particle sizes, with limitations for analyzing particles smaller than 100 nm, the combination of two different and complementary techniques, such as single-particle analyzers (NTA, DLS, high-resolution flow cytometry) with EM-based analysis is highly recommended in order to better characterize fungal EVs (Thery et al., 2018; Margolis and Sadovsky, 2019).

Methods of dehydration or chemical fixation for conventional EM pose many questions regarding artifacts and the possibility of altered morphology, size, and membrane stability of EVs (Noble et al., 2020). In this sense, the use of cryo-electron microscopy seems to be an appropriate method to visualize a broad spectrum of sizes and morphologies of EVs (Emelyanov et al., 2020; Noble et al., 2020). The use of asymmetric-flow field-flow fractionation for EV analysis is promising for the identification of larger and smaller vesicles (Zhang et al., 2018), but this remains to be confirmed in the analysis of fungal EVs. It is also important to state that linking size information to other biophysical and biochemical EV properties is of high relevance in order to define the vesicle subtypes better (Thery et al., 2018; Margolis and Sadovsky, 2019).

Clearly, there are still many open questions regarding the biological relevance of EV size diversity (Margolis and Sadovsky, 2019). Yet, novel approaches to optimize EV purification and size-based fractionation are needed. If resolved, this experimental limitation would facilitate the analysis of the impact of external factors on EV production, including growth conditions, cell cycle stage, growth phase, and cellular density, among others. In association with the optimized EV purification protocols, improved methods of data interpretation could positively impact the field.

For instance, comparative analyses of the enrichment of selected sets of molecules in EVs with their global cellular levels could reveal the existence of specific sorting mechanisms of molecular loading, in addition to selective delivery of EVs to different cell targets. Accordingly, it was recently shown that the claudin-like Sur7 family proteins Sur7 and Evp1 were enriched in *C. albicans* EVs, compared to whole cell lysates. The authors suggested these proteins as putative *C. albicans* EV positive markers, based on their potential topological similarity to tetraspanins, markers used for mammalian EVs (Dawson et al., 2020).

**Figure 2:**
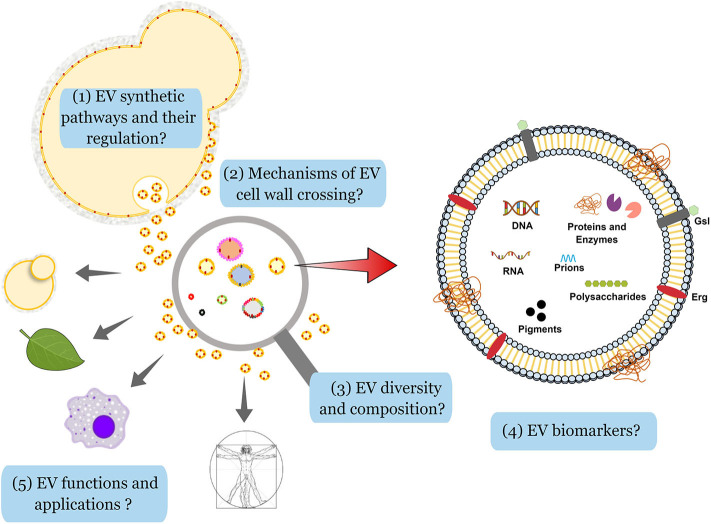
Challenges in the analysis of fungal EVs. By analogy with the metazoans, and based on the knowledge on secretion and regulation of vesicle production in eukaryotes, one can anticipate the existence of a least two pathways for the production of EVs in fungi. Although some elements implicated in these pathways (ESCRT proteins, for instance) have been already identified in fungi, little is known about the differential regulation in the formation of fungal EVs (1). In fungi, the presence of a thick cell wall was initially considered as a physical barrier for EV crossing, but recent evidence has shown that the cell wall is a viscoelastic structure and different hypotheses have been proposed to explore how it is compatible with the transit of lipid membranes (2). On the experimental side, a large diversity of EV morphology, size, and content has been observed, but different technical limitations have impaired the analysis. For instance, NTA or flow cytometry analyses do not detect particles smaller than 100 nm in diameter in a reliable fashion. Moreover, the current protocols used to purify EVs do not prevent contamination with aggregates potentially containing proteins, nucleic acids and polysaccharides, thus limiting the knowledge on EV structure and cargo. Differential EV purification according to their size and/or density from the contaminating aggregates should shed light on their intrinsic diversity (3). Despite their complex composition, including nucleic acids, glycans, pigments, proteins, prions, and different lipids (ergosterol, Erg; glycosphingolipids, Gsl), specific markers of fungal EVs have not been identified so far (4). Finally, very little is known on the role fungal EVs in cell-to-cell communication between different hosts and fungi, but also between fungal cells. One can anticipate that EV diversity could be associated with a diversity of functions and responses of the recipient cells in the presence of different types of vesicles. EV components may also have applicability in biomedical fields, including immunomodulation tools, drug-delivery vehicles, and vaccine candidates (5).

The analysis of cargo and the functional diversity of EVs is highly dependent on the purification methods and the nutritional availability (Tkach et al., 2018; Cleare et al., 2020). It was also demonstrated that EV-mediated prion export is regulated by glucose availability in *S. cerevisiae* (Kabani et al., 2020). Additionally, recent data showed that different nutrition environments play an essential role in EV formation and cargo loading in *H. capsulatum* (Cleare et al., 2020). Variable nutrient availability impacted the released EVs in size, protein, lipid, and carbohydrate metabolites profiles, which reinforce the plasticity of EV composition and its possible impact on fungal virulence (Cleare et al., 2020). Therefore, improvement of existing purification protocols and standardization of data analysis are highly desirable, as previously discussed (Coumans et al., 2017; Mateescu et al., 2017; Thery et al., 2018; Srinivasan et al., 2019; Thane et al., 2019; Turchinovich et al., 2019). The main challenges faced by the fungal EVs community, based on the technical hurdles and conceptual gaps described in this manuscript, are summarized in Figure 2.

## EVs as Vehicles for the Delivery of Biologically Active Molecules

There is an urgent need for new strategies to prevent and combat fungal infections, which affect over a billion people worldwide and kill more than 1.5 million, annually (GAFFI; http://www.gaffi.org/). Moreover, it is important to emphasize that the emergence of new fungal pathogens and resistance to antifungal drugs are health security threats around the world (Fisher et al., 2018; Rhodes and Fisher, 2019). Despite this public health burden, fungal infections have been widely neglected in terms of research funding compared to other infectious diseases (Rodrigues and Albuquerque, 2018; Rodrigues and Nosanchuk, 2020). In this sense, investments in combating fungal pathogens, including EVs-based approaches, can be beneficial for the development of new mechanisms of fighting fungal diseases.

Despite the already mentioned open questions (Margolis and Sadovsky, 2019; Raposo and Stahl, 2019), substantial progress has been made to elevate EVs to the position of important mediators of intercellular and interkingdom communication processes, given their ability to transfer bioactive components (Maas et al., 2017; van Niel et al., 2018; Cai et al., 2019; Mathieu et al., 2019; Correa et al., 2020) and surmount biological barriers, including the blood-brain barrier (BBB) (Alvarez-Erviti et al., 2011).

Potential EV-based applied tools are increasing in number and they are expected to positively affect diagnosis and therapy in a number of diseases (Lane et al., 2018; Shah et al., 2018). Their use as disease biomarkers, drug delivery, or even bioengineered for vehicles in therapeutics has also been proposed (Shah et al., 2018; Xu et al., 2018; Wiklander et al., 2019). In a sense, bacterial, fungal and parasite RNA sequences were identified in human body fluids (Beatty et al., 2014), suggesting that RNA-associated EVs could represent biomarkers of infectious diseases (Beatty et al., 2014; Hoy et al., 2014). EV-based strategies for the control of infectious diseases have also been suggested, including the use of artificial vesicle-protected RNA antifungal strategies, and other RNA-based techniques for host-induced gene silencing (Cai et al., 2019).

EVs have also been suggested to have important functions as adjuvants, vaccine or immunotherapy platforms for fighting infectious diseases, including fungal infections (Schorey and Harding, 2016; Fuhrmann et al., 2017; Kuipers et al., 2018; Freitas et al., 2019). In bacteria, for instance, *Staphylococcus aureus* EVs have been recently suggested to be used as a vaccine platform, since mice immunized with native *S. aureus* EVs produced a robust T-cell response and were protected against lung infection (Choi et al., 2015; Wang et al., 2018). Vaccine candidates for parasitic helminths are also being discussed since no effective vaccines are available to control the transmission of these pathogens (Mekonnen et al., 2018). Similarly, there are no licensed antifungal vaccines (Nami et al., 2019), which highlights the need for novel strategies to develop vaccines to prevent fungal infections.

Previous studies using EVs released by *C. neoformans* and *C. albicans* revealed their ability to delay the mortality of *Galleria mellonella* after challenge with yeast cells (Vargas et al., 2015; Colombo et al., 2019), probably due to an innate mechanism of infection control (Freitas et al., 2019). Considering that fungal EVs are efficient immunomodulators (Freitas et al., 2019), it is reasonable to suggest that native or engineered EVs could be promising structures for the development of vaccine platforms.

## Concluding Remarks

Fungal EVs have been the focus of many studies over the past few years and have emerged as important signaling particles, therefore opening new avenues for investigation of their use in different pathogenic models. Despite substantial advances in the field, important challenges and unanswered questions about the structure and functions of EVs remain active. There is a clear need to strengthen our knowledge of the genetic, biochemical, and physical aspects of fungal EVs in order to clarify the mechanisms regulating their production, composition, and diversity toward a better comprehension of their biological function.

## Author Contributions

JR contributed to the conception and design of the review. MR and GJ contributed equally to the manuscript text and revision. MR was currently on leave from his position of Associate Professor in the Instituto de Microbiologia Paulo de Góes, Universidade Federal do Rio de Janeiro (UFRJ), Brazil. All authors have made substantial intellectual contributions to the work, have read, and approved the submitted version.

## Conflict of Interest

The authors declare that the research was conducted in the absence of any commercial or financial relationships that could be construed as a potential conflict of interest.
